# *In silico *identification of genes involved in selenium metabolism: evidence for a third selenium utilization trait

**DOI:** 10.1186/1471-2164-9-251

**Published:** 2008-05-29

**Authors:** Yan Zhang, Anton A Turanov, Dolph L Hatfield, Vadim N Gladyshev

**Affiliations:** 1 Redox Biology Center and Department of Biochemistry, University of Nebraska, Lincoln, NE 68588, USA; 2 Section on the Molecular Biology of Selenium, Laboratory of Cancer Prevention, Center for Cancer Research, National Cancer Institute, National Institutes of Health, Bethesda, MD 20892, USA

## Abstract

**Background:**

Selenium (Se) is a trace element that occurs in proteins in the form of selenocysteine (Sec) and in tRNAs in the form of selenouridine (SeU). Selenophosphate synthetase (SelD) is required for both utilization traits. However, previous research also revealed SelDs in two organisms lacking Sec and SeU, suggesting a possible additional use of Se that is dependent on SelD.

**Results:**

In this study, we conducted comparative genomics and phylogenetic analyses to characterize genes involved in Se utilization. Candidate genes identified included SelA/SelB and YbbB that define Sec and SeU pathways, respectively, and NADH oxidoreductase that is predicted to generate a SelD substrate. In addition, among 227 organisms containing SelD, 10 prokaryotes were identified that lacked SelA/SelB and YbbB. Investigation of *selD *neighboring genes in these organisms revealed a SirA-like protein and two hypothetical proteins HP1 and HP2 that were strongly linked to a novel Se utilization. With these new signature proteins, 32 bacteria and archaea were found that utilized these proteins, likely as part of the new Se utilization trait. Metabolic labeling of one organism containing an orphan SelD, *Enterococcus faecalis*, with ^75^Se revealed a protein containing labile Se species that could be released by treatment with reducing agents, suggesting non-Sec utilization of Se in this organism.

**Conclusion:**

These studies suggest the occurrence of a third Se utilization trait in bacteria and archaea.

## Background

Selenium (Se) is an essential micronutrient for many organisms in the three domains of life. The best known biological functions of Se are exerted by selenocysteine (Sec) residues [1-3]. Sec, known as the 21^st ^amino acid, is co-translationally inserted into proteins by recoding an opal (UGA) codon from stop to Sec function [4-6]. These UGA codons are recognized by a complex molecular machinery that has common core components, but also differences among the three domains of life [3,4,7-10].

The mechanism of Sec insertion into protein in response to UGA has been most thoroughly elucidated in *Escherichia coli *[3,4,11-13]. Bacterial selenoprotein mRNAs carry a Sec insertion sequence (SECIS) element immediately downstream of Sec-encoding UGA codons [4,5]. The SECIS element binds a Sec-specific elongation factor, SelB, and forms a complex with tRNA^Sec ^(SelC), whose UCA anticodon matches the UGA codon. tRNA^Sec ^is initially acylated with serine by seryl-tRNA synthetase and is then converted to Sec-tRNA^Sec ^by Sec synthase (SelA). SelA utilizes selenophosphate as the selenium donor, which in turn is synthesized by selenophosphate synthetase (SelD).

In some prokaryotes, Se (also in the form of selenophosphate) is used for the biosynthesis of a modified tRNA nucleotide, 5-methylaminomethyl-2-selenouridine (mnm^5^Se^2^U, or SeU), which is located in the wobble position of the anticodons of tRNA^Lys^, tRNA^Glu^, and tRNA^Gln ^[14-16]. The proposed function of SeU involves codon-anticodon interactions that help base pair discrimination at the wobble position and/or translation efficiency [16,17]. A 2-selenouridine synthase (YbbB) is necessary to replace a sulfur atom in 2-thiouridine in these tRNAs with Se [18].

In addition to Sec and SeU, Se can be utilized in the form of a cofactor in certain molybdenum (Mo)-containing hydroxylases [19-23]. Nicotinic acid hydroxylase and xanthine dehydrogenase are the best known representatives of this protein class. In these enzymes, Se is covalently bound to Mo in the active site, but the specific structure of the Se cofactor is not known. In nicotinic acid hydroxylase, Se is lost during protein storage and during simple SDS-PAGE procedures [20]. These properties made it difficult to characterize this class of proteins and determine the mechanism of Se cofactor structure and biosynthesis.

Recently, we analyzed evolutionary dynamics of Sec and SeU utilization traits (i.e., analyzed genes involved in the corresponding biosynthetic pathways) in prokaryotes and reported the occurrence of orphan SelD proteins in two organisms lacking known components of Sec and SeU traits or genes encoding selenoproteins [24]. These organisms included a bacterium, *Enterococcus faecalis*, and an archaeon, *Haloarcula marismortui*. The SelD sequences in the two organisms are typical SelDs containing a conserved Cys residue in the predicted active site and clustering with other SelD sequences [24]. These proteins could be distinguished from thiamine-monophosphate kinase, the hydrogenase maturation factor HypE, and other proteins that, like SelDs, have an aminoimidazole ribonucleotide synthetase (AIRS) domain. The curious presence of orphan SelD in these prokaryotes suggested an additional, unknown use of Se that is dependent on SelD.

In this study, we carried out searches in completely sequenced prokaryotic genomes for machinery involved in Se utilization. Known components, i.e., SelA, SelB and YbbB, could be easily identified by comparative genomics, and the analyses also have generated evidence for additional proteins involved. Since neighboring genes of *selD *may provide potential information regarding Se utilization, we further employed comparative genomics tools to identify candidate genes involved in the third, SelD-based trait, in prokaryotes. Finally, we identified many organisms containing this new trait and carried out experimental analyses in one such organism. Overall, these data provide evidence for an additional use of Se in nature.

## Results and Discussion

### Characterization of SelD-dependent pathways of Se utilization

Since SelD (COG0709) has been shown to be a key factor for Se utilization by generating a Se donor compound (and therefore discriminating Se from sulfur for further use), identification of functional linkages involving SelD may help characterize the pathways of Se utilization. We initially used STRING [[25], the interactor was set to COG-mode] to examine such functional linkages based on neighborhood, gene fusion and co-occurrence analyses. The protein with the best score was YbbB, a SeU synthase (COG2603). This gene was often located in the same operon with *selD *and the two proteins also showed similar patterns of occurrence [24]. The next SelD link was SelB (COG3276), which was also identified by gene neighborhood and co-occurrence, but the linkage was independent of YbbB (because *selD *formed operons with either *ybbB *or *selB *but rarely with both). As expected, SelB was most closely linked with Sec synthase SelA (COG1921), which also showed a strong association with SelD.

The following SelD link was NADH oxidoreductase homolog (COG1252), which was fused with SelD in cyanobacteria and several other organisms [24]. This association suggests that NADH oxidoreductase may be the reductant for a Se compound and that the reduced form of this compound may be utilized by SelD for biosynthesis of selenophosphate. Like YbbB and SelA/SelB, NADH oxidoreductase function likely corresponds to the known use of Se. Excluding spurious predictions due to protein misannotation, the following hit was a SirA-like protein that belonged to COG0425 (predicted redox protein, regulator of disulfide bond formation). This protein was associated with SelD through gene neighborhood (i.e., location of genes next to each other, or in close vicinity, in the genome) predictions of STRING. Thus, STRING-based analysis identified known proteins involved in two pathways of Se utilization (e.g., SelA/SelB in the Sec pathway, and YbbB in the SeU pathway) and suggested the role of NADH dehydrogenase in generating a SelD substrate and of SirA-like protein in an unknown SelD-linked process.

### Organisms with orphan SelDs

**Figure 1:**
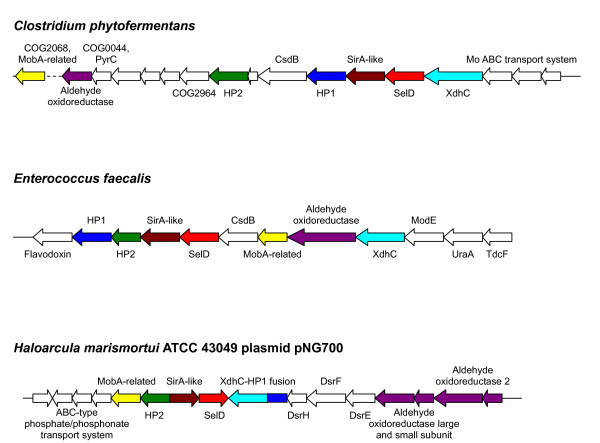
**Genomic context of *selD *in completely sequenced genomes containing orphan SelD**. Candidate genes in the completed genomes of *Clostridium phytofermentans*, *Enterococcus faecalis *and *Haloarcula marismortui *ATCC 43049 plasmid pNG700 are color coded. Coding direction is also indicated.

Next, we examined SelD-linked processes in more detail, and in particular identities of SelD-linked genes that showed no association with the Sec and SeU pathways. Among 589 sequenced prokaryotic genomes, we identified 227 SelD-containing organisms (219 bacteria and 8 archaea). Details are shown in Table S1 [see Additional file 1]. Of these, 140 bacteria and 7 archaea possessed the Sec pathway, whereas 148 bacteria and 6 archaea utilized SeU (the two traits partially overlapped). In addition, 10 SelD-containing organisms were found that had orphan SelD (i.e., they had SelD but lacked both SelABC and YbbB), including previously described *E. faecalis *and *H. marismortui*. Additional such organisms included *Anaerostipes caccae*, *Clostridium butyricum*, *C. phytofermentans*, *Faecalibacterium prausnitzii*, *Ruminococcus gnavus*, *R. obeum*, *R. torques *and *Vibrio shilonii*. It should be noted that except for *E. faecalis*, *H. marismortui *and *V. shilonii*, all other species belonged to *Firmicutes/Clostridia *where many species possess Sec and/or SeU utilization traits. However, previous studies have shown a highly dynamic evolution of both traits, which often results in the loss of these traits in bacterial phyla [24]. Moreover, of the 10 organisms with orphan SelDs, *C. phytofermentans*, *E. faecalis *and *H. marismortui *have been completely sequenced, and most other genomes are characterized by high sequence coverage (e.g., 9.8× for *A. caccae*, 8.53× for *C. butyricum *8.7× for *R. gnavus*, 8.9× for *R. obeum *and 11.6× for *R. torques*). In addition, *selD *typically clusters with Sec and/or SeU biosynthesis/insertion genes, whereas in these organisms, other genes cluster with *selD*. Thus, the possibility that all genes involved in Sec or SeU utilization have not been sequenced or annotated in the genomes containing orphan SelD is extremely low. The genomic context of *selD *in the three complete orphan SelD-containing genomes is shown in Fig. 1. Genomic context of the other 7 organisms is shown in Fig. S1 [see Additional file 2].

### A third pathway of Se utilization

To investigate a possible new SelD-associated pathway, 10 genes upstream and downstream of *selD *in the genomes with orphan SelDs were examined in detail. First, we found that the *sirA-like *gene (EF2566, SirA-like domain is located in the N-terminal of the protein, the C-terminal domain is a distant homolog of DsrE family) is located next to *selD *in several organisms in different bacterial phyla, suggesting that the SirA-like protein may be involved in Se metabolism. This observation is consistent with the STRING analysis. The specific function of SirA-like is not clear.

**Figure 2:**
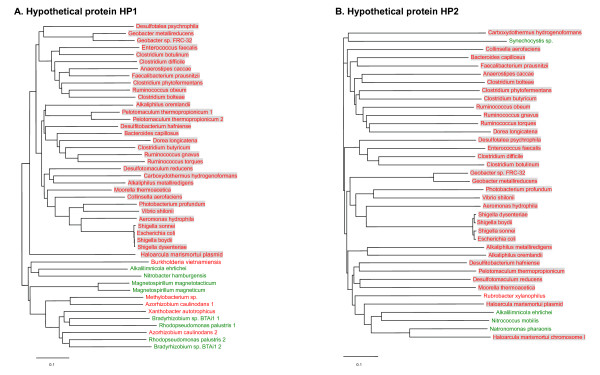
**Phylogenetic analysis of HP1 and HP2**. SelD-containing and SelD-lacking organisms are shown in red and green, respectively. Organisms which contain SelD, HP1 and HP2 are shaded. (A) Hypothetical protein HP1. (B) Hypothetical protein HP2.

**Figure 3:**
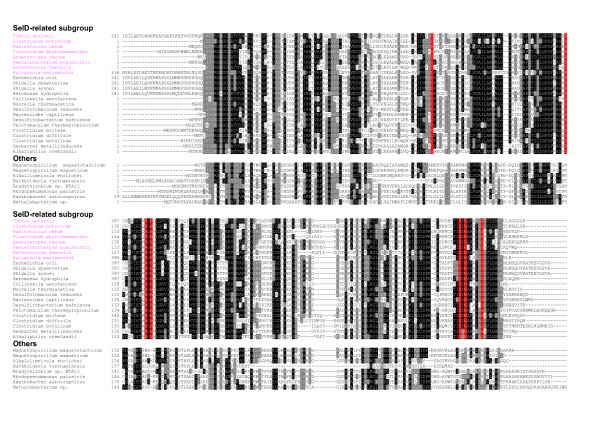
**Multiple alignment of hypothetical protein HP1**. Representative sequences were divided into two groups: SelD-related subgroup and other homologs. Residues which are strictly conserved in the SelD-related subgroup are shown in red background. Other residues shown in white on black or grey are conserved in homologs. Organisms containing orphan SelD are highlighted in pink font.

Next, we found that two additional proteins, hypothetical proteins HP1 (EF2563) and HP2 (EF2564), co-occurred in SelD-containing organisms, including all orphan SelD-containing organisms, although homologs of each protein could also be found in organisms lacking SelD. Phylogenetic analyses of these two proteins are shown in Fig. 2. A total of 32 organisms (14% of all SelD-containing organisms, most are Firmicutes/Clostridia) containing both HP1 and HP2 proteins as well as SelD were identified. We noticed that all HP1 sequences in organisms having SelD and HP2 were clustered in one subfamily, suggesting that these sequences might be functionally linked to SelD. Orthologs of HP1 were also found in additional SelD-containing organisms (e.g., *Burkholderia vietnamiensis *and *Azorhizobium caulinodans*) which did not have HP2. Although significant similarity was observed between sequences in this putative subfamily and other homologs (e.g., e-value is 8e-13 and identity is over 33% between *E. faecalis *and *B. vietnamiensis*), multiple alignment of HP1 sequences suggested several specific residues which are only present in the SelD-linked subfamily (Fig. 3). Therefore, it appears that these HP1 proteins form a separate subfamily which is involved in the third Se utilization trait, perhaps distinguished by some of these conserved residues. In addition, most HP2 sequences were found in organisms containing both SelD and HP1, except for 5 organisms which lacked HP1 (4 SelD-lacking and 1 SelD-containing). Previously, we observed that homologs of SelA, a key factor in Sec biosynthesis in bacteria, are also found in organisms that lack the Sec-decoding trait, suggesting that SelA (or its close homologs) might have acquired a new function in these organisms [24]. Similarly, HP1 or HP2 homologs may also have additional functions in organisms lacking SelD. Co-occurrence of SelD, HP1 and HP2 might provide an initial screen for identifying organisms with additional utilization of Se. Although two thirds of these organisms also possess either Sec or SeU utilization traits, the fact that 10 out of 32 organisms belonging to three different phyla (Firmicutes/Clostridia, Firmicutes/Lactobacillales and Proteobacteria/gamma/Vibrionaceae) possess orphan SelDs argues against the possibility that these organisms are simultaneously in the process of acquiring the Sec or SeU traits or of losing such traits.

Other candidate genes in the vicinity of *selD *in *E. faecalis *genome, e.g., EF2565 (Hypothetical protein), EF2568 (COG0520, CsdB) and EF2572 (ModE), showed wider distribution than SelD itself. However, some members of these families were *selD *neighbors in several organisms, and some were even fused with HP1 or HP2. These included EF2569 (a MobA-related protein), EF2570 (this protein is annotated as aldehyde oxidoreductase which belongs to xanthine oxidase family, and contains both [2Fe-2S]-binding and molybdopterin-binding domains) and EF2571 (COG1975, XdhC, Xanthine and CO dehydrogenases maturation factor, XdhC/CoxF family). These observations suggest functional links among these proteins. Table 1 shows the genomic location of all candidate genes (including *selD *and *sirA-like*) in the 32 organisms defined by HP1, HP2 and SelD proteins. Although *selD *was found to be in vicinity of HP1 and HP2 genes only in 5 organisms, all of them were orphan SelD-containing prokaryotes.

**Table 1:** Genomic locations of candidate Se utilization genes in organisms containing the third Se utilization trait

**Phylum**	**Organisms**	**SelD**	**SirA-like**	**HP1**	**HP2**	**COG2068 (MobA-related)**	**XdhC**	**Aldehyde oxidoreductase (Mo-binding subunit)**
Actinobacteria	Collinsella aerofaciens	*Cont1020: 232592–233626^1^	*Cont1020: 231905–232531	**Cont930: 22536–23330	**Cont930: 21699–22433	**Cont930: 19598–20212	**Cont930 23340–24383	Cont981 271699–274452 Cont1020 200889-198112
Bacteroidetes	Bacteroides capillosus	Cont352: 18981–20015	Cont176: 77167–77790	*Cont29: 35523–36383	*Cont29: 36383–37171	*Cont29: 30639–31217	*Cont29: 34780–35535	Cont138: 59682–62240
Firmicutes/Clostridi	Alkaliphilus metalliredigens	2387682– 2388704	40091–40726	*4677649–4678437	*4679824–4680603	*4679235–4679813	*4678431–4679234	722641–724956, *4682345–4683322
a	Alkaliphilus oremlandii	1841596–1842636	27112–27720	2083961–2084752	*432911–433723	*433705–434277	2277921–2278991	*428856–430112
	***Anaerostipes caccae***^3^	*Cont17: 40772–41797	*Cont17: 40136–40756	**Cont9: 91090–91908	**Cont9: 78202–78897	**Cont9: 65080–65661	**Cont9: 65664–66623	**Cont9: 93298–95865, Cont17: 103961–106198
	Carboxydothermus hydrogenoformans	1842765–1843796	440696–441091	*607679–608986(F)^2^	*607002–607679	*607679–608986(F)	1802422–1803132	*604273–605949
	Clostridium bolteae	Cont239: 58918–59949	Cont5: 241032–241658	Cont308: 91210–92058	*Cont308: 51808–52677	*Cont308: 51201–51791	*Cont308: 53226–54257	Cont239: 11354–13936 Cont308: 83751–86114
	Clostridium botulinum	3144455–3145543	2625432–2626016	*3014560–3015408	*3013213–3013950	*3013965–3014579	*3015390–3016199	*2996596–2999151
	***Clostridium phytofermentans***	*1829925–1830947	*1831098–1831769	*1831953–1832798	*1834253–1834996	1868957–1869568	*1828657–1829781	1841927–1844488
	Clostridium difficile 630	2880768–2881796	4276359–4276961	4066403–4067218	*2386592–2387314	*2385916–2386494	1808813–1809634	3713531–3716092, 2408777–2410993
	***Clostridium butyricum***	*NZ_ABDT01000004: 155650–156687	*NZ_ABDT01000004: 156793–157389)	*NZ_ABDT01000004: 158639–159460	*NZ_ABDT01000004: 159523–160266	*NZ_ABDT01000004: 149490–150173	*NZ_ABDT01000004: 157568–158620	*NZ_ABDT01000004: 150163–152445
	Desulfitobacterium hafniense	*5324753–5325775	*5323991–5324584	**1050540–1051346	**1049119–1049871	**1049868–1050500	**1063079–1064179, 2283203–2284381	2292035–2294767, **1054910–1057801
	Desulfotomaculum reducens	*1315312–1316340	*1316345–1316935	**328528–329328	***2996229–2996984	***2995639–2996232	**329303–330112	1631845–1634121, ***3003610–3005700
	Dorea longicatena	*Cont11: 5103–6131	*Cont11: 4318–4923	**Cont1: 48159-47407(F)	**Cont1: 48938-48294	**Cont1: 48159-47407(F)	Cont390: 11270–12310	Cont429: 382570–384858
	***Faecalibacterium prausnitzii***	*Cont742: 186688–187725	*Cont742: 187722–188354	*Cont742: 190792–191607	*Cont742: 185990–186691	*Cont742: 181661–182344	*Cont742: 189740–190795	*Cont742: 183695–185983
	Moorella thermoacetica	1661283–1662269	121583–122164	*2088495–2089295	*2089404–2090771(F)	*2089404–2090771(F)	*2091852–2092601	*2083615–2086176
	***Ruminococcus gnavus***	*Cont378: 107582–108643	*Cont378: 106942–107568	**Cont46: 121015–122373(F)	**Cont46: 120257–121018	**Cont46: 121015–122373(F)	Cont378: 183010–183981	**Cont46: 122404–124974, Cont378: 179128–181425
	***Ruminococcus obeum***	Cont14: 66952–67983	Cont266: 10819–11544	*Cont16 33528–34331	*Cont16 34380–35003	*Cont16: 35054–35641	Cont159: 118818–119843	*Cont16: 27798–29306
	***Ruminococcus torques***	*Cont155: 61958–63010	*Cont155: 66853–67602	**Cont52 237566–238321(F)	**Cont52 236865–237479	**Cont52 237566–238321(F)	**Cont52: 230688–231806	Cont63: 18998–21319
	Pelotomaculum thermopropionicum	1934961–1935986	1568109–1568714	*1602214–1603053, 1623719–1624549	*1601464–1602207	*1600834–1601427	679591–680640, *1603053–1604195	*1607123–1609426, 1657476–1659872
Firmicutes/Lactobacillales	***Enterococcus faecalis***	*2481426–2482448	*2480823–2481410	*2479008–2479802	*2479802–2480557	*2484207–2483629	*2486915–2487922	*2484221–2486794
Proteobacteria/delta	Desulfotalea psychrophila	*1085079–1086110	*1084463–1085125	2105056–2105937	**3464087–3464815	**3463504–3464127	282635–283651	2882406–2884739, **3464796–3467099 290004–292772,
	Geobacter metallireducens	3290478–3291509	1209663–1210253	*2405213–2406019	*2406030–2406812	*2406812–2407420	*2404445–2405206	*2402117–2404423
	Geobacter sp. FRC-32	ctg246: 23921–24949 ctg184: 10957–11982	ctg221: 1602–2192	*ctg252 9051–10067	*ctg252 8261–9046	*ctg252 7537–8190	*ctg252 9862–10623	*ctg252: 10761–13064
Proteobacteria/gamma/Entero bacteriales	Escherichia coli	1844989–1846032	2082250–2082477 (distant)	*3010636–3012261(F)	*3012309–3013079	*3013182–3013760	*3010636–3012261(F)	2998367–3000625, *3019338–3022208
	Shigella sonnei	1465531–1466574	2176848–2177075 (distant)	*3182128–3183753(F)	*3183801–3184508	*3184674–3185252	*3182128–3183753(F)	*3170040–3172298, *3193619–3196474
	Shigella boydii	1307704–1308747	852439–852666 (distant)	*3110074–3111699(F)	*3109319–3110026	*3108575–3109153	*3110074–3111699(F)	3121932–3124190
	Shigella dysenteriae	Sdys1_01_12: 44728–45771	Sdys1_01_2: 245061–245288	*Sdys1_01_9: 31086–32711(F)	*Sdys1_01_9: 32759–33529	-	*Sdys1_01_9: 31086–32711(F)	*Sdys1_01_9: 23377–25674
Proteobacteria/gam ma/Vibrionaceae	Photobacterium profundum	1695833–1696864	59336–59584 (distant)	*2279625–2281223(F)	*2281263–2282165	*2282226–2282801	*2279625–2281223(F)	*2235899–2238079, *2222702–2225572
	***Vibrio shilonii***	*1103207001845: 47718–48761	1103207001999: 14643–14870 (distant)	**1103207001836 24519–26117(F)	**1103207001836 23687–24496	**1103207001836: 23042–23635	**1103207001836 24519–26117(F)	*1103207001845: 42086–45310 **1103207001836: 19080–21950
Proteobacteria/gamma/Others	Aeromonas hydrophila	2454023–2455060	1829442–1829684 (distant)	*2377424–2379016(F)	*2379036–2379884	*2379865–2380518	*2377424–2379016(F)	2394101–2396419 *2386262–2389159
Archaea	***Haloarcula marismortui plasmid pNG700***	*247358–248011	*246720–246968 (distant)	*249609–248032(F)	*245843–246625	*245179–245796	*249609–248032(F)	*253850–256300, *250677–252842

**Notes**: 1. *, ** or ***, Genes which are located in different operons

2. (F), Fusion genes

3. Organisms containing orphan *selD *gene are shown in bold and italic.

**Figure 4:**
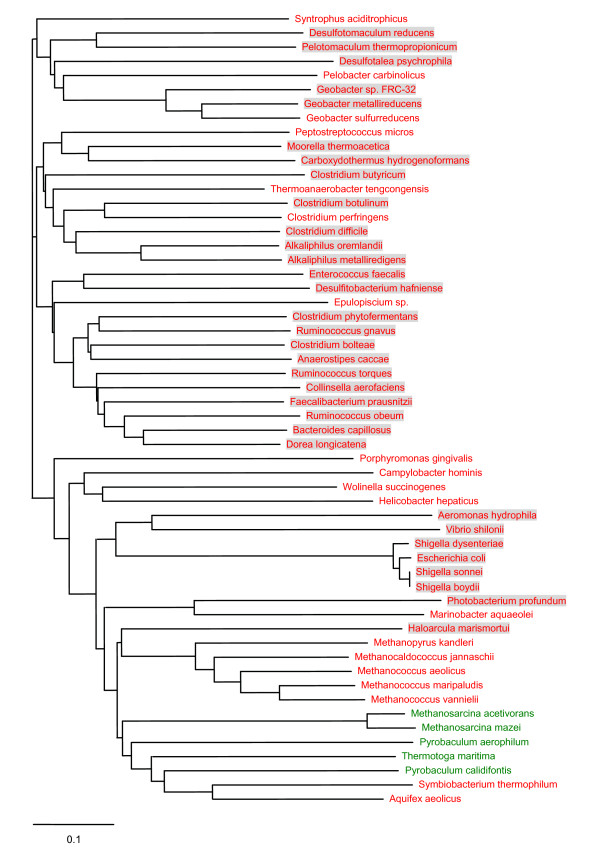
**Phylogenetic analysis of SirA-like proteins**. SelD-containing and SelD-lacking organisms are shown in red and green, respectively. Organisms which contain SelD, HP1 and HP2 are shaded.

**Figure 5:**
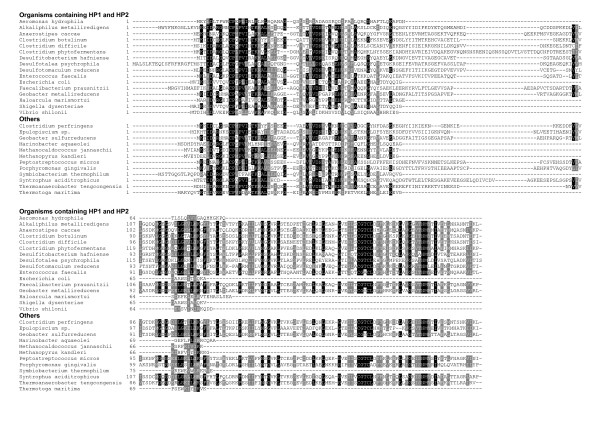
**Multiple alignment of SirA-like proteins**. Representative sequences were divided into two groups: organisms containing HP1 and HP2 and other homologs. Residues shown in white on black or grey are conserved in homologs.

The SirA-like protein was also found in all 32 organisms possessing HP1 and HP2 proteins although some of these organisms contained its slightly more distant homologs (e.g., *H. marismortui *and *E. coli*). As discussed above, *selD *is often clustered with *sirA-like*, and this situation was found in 12 out of 32 organisms. Close homologs of SirA-like protein were also detected in several organisms lacking HP1 and HP2, such as *Methanococcus maripaludis *(Sec-utilizing archaea), *Clostridium perfringens *and *Thermoanaerobacter tengcongensis *(Sec-utilizing bacteria) and *Porphyromonas gingivalis *(SeU-utilizing bacteria). Considering that proteins containing SirA domain belong to a large superfamily and may have different functions, phylogenetic analysis was used to identify a SirA subfamily most closely linked to HP1 and/or HP2 (Fig. 4). However, no such branch was found. Moreover, multiple alignment of SirA-like sequences did not show conserved residues which are only present in organisms containing HP1 and HP2 (Fig. 5).

To further examine functional linkages among SelD, SirA-like, HP1 and HP2 proteins, we, again, used STRING (neighborhood and co-occurrence tools) and searched for functional associations involving SirA-like, HP1 and HP2. Since no known COG is associated with HP1 or HP2, and SirA-like belongs to a large family of SirA proteins defined by a single COG, we selected a "search by protein sequence" option and set the interactor tool to a protein mode. Such configuration can provide maximum sensitivity although it has a slightly lower coverage compared with the COG mode [25]. Top candidates are shown in Table 2, and SelD was among these candidates for each examined protein. In addition, SelD was the top functional link for SirA-like. Although both SirA-like and HP1/HP2 seem to be functionally linked to SelD, there are important differences among these proteins and which of these proteins best define the putative third Se utilization trait is not clear. It is possible that SirA-like protein is involved in Se metabolism in all three utilization traits whereas HP1 and HP2 are only specific for the third Se utilization trait.

**Table 2:** STRING analysis of genes functionally associated with SirA-like, HP1 and HP2 in *E. faecalis*

**Rank**	**SirA-like (EF2566)**	**HP1 (EF2563)**	**HP2 (EF2564)**
1	EF2567 (SelD)	EF2564 (HP2)	EF2563 (HP1)
2	EF2568 (Aminotransferase, class V)	EF2569 (MobA-related protein)	EF2565 (Hypothetical protein)
3	EF2564 (HP2)	EF2570 (Aldehyde oxidoreductase, putative)	EF2569 (MobA-related protein)
4	EF2563 (HP1)	EF2565 (Hypothetical protein)	EF2566 (SirA-like)
5	EF2565 (Hypothetical protein)	EF2566 (SirA-like)	EF2578 (Peptidase, M20/M25/M40 family)
6	EF2570 (Aldehyde oxidoreductase, putative)	EF2578 (Peptidase, M20/M25/M40 family)	EF2567 (SelD)
7	EF2569 (MobA-related protein)	EF2577 (Aspartate/ornithine carbamoyltransferase family)	EF2570 (Aldehyde oxidoreductase, putative)
8	EF1394 (Hypothetical protein)	EF2567 (SelD)	EF2568 (Aminotransferase, class V)
9	EF2562 (Flavodoxin)	EF2571 (XdhC)	EF2577 (Aspartate/ornithine carbamoyltransferase family)
10	EF2571 (XdhC)	EF2568 (Aminotransferase, class V)	EF2562 (Flavodoxin)

Several other detected *selD *neighboring genes were often located in the same operon. One exception was that we could not detect homologs of MobA-related protein (COG2068) in *Shigella dysenteriae*. However, this genome is not yet complete. Previous studies showed that some of these genes are involved in the formation and utilization of molybdopterin (MPT), which coordinated Mo thereby generating the Mo cofactor in Mo-dependent enzymes [26-28]. For example, XdhC is present in various Mo-utilizing organisms and is involved in Mo cofactor binding and insertion into xanthine dehydrogenase [29]. It is possible that Se (in the form of selenophosphate) is used as an additional cofactor that supports Mo utilization in certain organisms. The predicted aldehyde oxidoreductase in *E. faecalis*, which belongs to Mo-dependent xanthine oxidase family [27,28,30] and is often found to be clustered with HP1 and/or HP2 (see Table 1), might be a potential user which utilizes both Se and Mo. However, phylogenetic analysis of aldehyde oxidoreductase (both large and small subunit) did not yield a subfamily formed by organisms containing the new Se utilization trait (instead, they are scattered in different branches, data not shown).

### Metabolically labeled Se-binding protein in *E. faecalis*

**Figure 6:**
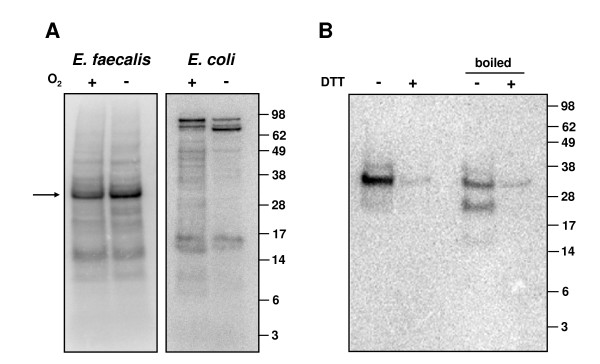
**Metabolic labeling of *E. faecalis *with ^75^Se and analysis of Se-binding proteins**. (A) *E. faecalis *and *E. coli *were metabolically labeled under aerobic and anaerobic conditions. 30 μg of total protein from each organism were resolved by SDS-PAGE gel and transferred onto a PVDF membrane. ^75^Se patterns were visualized with a PhosphorImager. Molecular masses of protein standards (in kilodaltons) are shown on the *right*. (B) Se-containing proteins were heated or treated with DTT. 30 μg of total protein from each organism were resolved by SDS-PAGE gel and transferred onto a PVDF membrane. ^75^Se patterns were visualized with a PhosphorImager. Molecular masses of protein standards (in kilodaltons) are shown on the *right*.

To directly analyze the use of Se in *E. faecalis*, we metabolically labeled this organism with ^75^Se under aerobic and anaerobic conditions, and in parallel we labeled *E. coli *strain Nova Blue cells as a control. In both conditions, a 30 kDa ^75^Se-labeled band was observed in *E. faecalis *extracts (SDS-PAGE in the absence of reducing agents), whereas as expected, *E. coli *showed bands in the 80–110 kDa region and the labeling pattern was different in aerobic and anaerobic conditions (Fig. 6A). After treatment with DTT (with or without heating), the band disappeared (Fig. 6B). 2-Mercatoethanol was also effective in releasing Se, whereas these treatments had no influence on the ^75^Se bands in *E. coli *extracts as these corresponded to Sec-containing proteins (data not shown). The observations suggest that *E. faecalis *does utilize Se, that this element occurs in a protein of ~30 kDa and that this Se species is labile and is not Sec. Therefore, it is possible that this 30 kDa protein may be involved in the third SelD-related, Se utilization trait. It should be noted, however, that an additional utilization of Se in *E. faecalis *unrelated to SelD function could not be excluded. Independent of its nature, both computational and experimental data exclude a possibility of Sec and SeU use and suggest a novel use of Se in this organism.

The large subunits of proteins in the xanthine oxidase family (COG1529, CoxL), whose molecular weights are over 80 kDa, bind MPT (27, 31, 32). The detected 30 kDa protein in *E. faecalis *is inconsistent with the aldehyde oxidoreductase large subunit. Similarly, the aldehyde oxidoreductase small subunit (CoxS), which binds 2Fe-2S, was also excluded because it is fused with the large subunit in *E. faecalis*. Thus, the 30 kDa Se-binding protein is unlikely to be a member of the Mo-dependent hydroxylase family. It is interesting, however, that both HP1 and HP2 have similar predicted molecular weights (28.5 kDa and 28.4 kDa, respectively). Thus, one of them might be the detected protein.

We carried out a number of chromatographic steps to purify the 30 kDa Se-binding protein. It was binding both DEAE-Sepharose and Phenyl-Sepharose and could be enriched on these columns. However, lability and consistent loss of Se precluded its identification. We also attempted two-dimensional PAGE analysis of the Se-binding protein (under non-reducing conditions), but no ^75^Se radioactive spot was found following this procedure, suggesting that two-dimensional PAGE conditions also led to the release of Se from the Se-binding protein. Further studies will be needed to determine the identity of the 30 kDa Se-binding protein. Our current studies, however, suggested the presence of a novel utilization of Se in an orphan SelD-containing organism, *E. faecalis*, which appears to be the third trait of Se utilization in prokaryotes.

## Conclusion

In this study, we carried out comparative genomics and phylogenetic analyses to identify new genes linked to Se utilization in prokaryotes. We identified several organisms with orphan SelD that we predict to possess the third Se utilization trait, which is not limited to these species. SelD, HP1, HP2 as well as SirA-like were identified as the best candidate signature genes for this trait. We further directly demonstrated the use of Se in *E. faecalis *by detecting a 30 kDa protein containing a non-Sec, non-SeU labile Se species. It cannot be excluded that Se is used as a co-factor for certain Mo hydroxylases (known to contain a labile Se cofactor), but current evidence does not provide strong support for this possibility. Further studies are required to determine whether the 30 kDa Se-binding protein or other proteins in organisms with orphan SelDs represent the use of Se in this organism, or it is an intermediate state for further delivery to other proteins, such as Mo-dependent hydroxylases.

## Methods

### Databases, genomes and sequences

Sequenced prokaryotic genomes from current Entrez Microbial Genome Project were used in this study (541 bacterial and 48 archaeal genomes; Feb 1, 2008). Due to the large number of sequenced strains for some bacterial species, we utilized only one strain from each species (e.g., *Escherichia coli K12 *represented all *Escherichia coli*).

We used *E. coli *SelA, SelB, SelD, and YbbB sequences as queries to search for components of Sec-decoding and SeU traits based on previously used criteria [24]. In addition, we selected 10 genes upstream and downstream of *selD *in the genomes with orphan SelDs. A list of *selD*-flanking genes in *E. faecalis *is shown in Table 3. For each of these proteins (including SelD), TBLASTN [33] was initially used to identify genes coding for homologs with a cutoff of E-value ≤ 0.01. Orthologous proteins were defined as bidirectional best hits [34]. When necessary, orthologs were also confirmed by genomic location analysis or building phylogenetic trees for the corresponding protein families.

**Table 3:** SelD-flanking genes in *E. faecalis *(sorted based on genomic location)

**Gene Locus Tag (*E. faecalis*)**	**Protein name**	**COG/Pfam (e-value < 0.1)**
EF2556	Fumarate reductase	COG1053, SdhA
EF2558	Cation transporter	COG0168, TrkG
EF2559	Pyruvate flavodoxin/ferredoxin oxidoreductase family protein	COG0674, PorA (N-terminal) COG1014, PorG (middle) COG1013, PorB (C-terminal)
EF2560	Glutamate synthase (NADPH), homotetrameric	COG0493, GltD
EF2561	Dihydroorotate dehydrogenase electron transfer subunit, putative	COG0543, UbiB
EF2562	Flavodoxin	COG0716, FldA
EF2563	Hypothetical protein HP1	-
EF2564	Hypothetical protein HP2	-
EF2565	Hypothetical protein	-
EF2566	SirA-like	COG0425, SirA (N-terminal)
EF2567	SelD	COG0709, SelD
EF2568	Aminotransferase, class V	COG0520, CsdB
EF2569	MobA-related protein	COG2068, Uncharacterized MobA-related protein
EF2570	Aldehyde oxidoreductase, putative	COG2080, CoxS (N-terminal) COG1529, CoxL (major part)
EF2571	Xanthine and CO dehydrogenases maturation factor	COG1975, XdhC
EF2572	Molybdenum transport domain protein ModE	COG2005, ModE
EF2573	Xanthine/uracil permease family protein	COG2233, UraA
EF2574	Endoribonuclease L-PSP, putative	COG0251, TdcF
EF2575	Carbamate kinase	COG0549, ArcC
EF2576	Hypothetical protein	-
EF2577	Ornithine carbamoyltransferase	COG0078, ArgF

### Multiple sequence alignment and phylogenetic analysis

Sequences were aligned with CLUSTALW [35] using default parameters. Ambiguous alignments in highly variable (gap-rich) regions were excluded. The resulting multiple alignments were then checked for conservation of functional residues and manually edited. Phylogenetic analyses were performed using PHYLIP programs [36]. Pairwise distance matrices were calculated by PROTDIST to estimate the expected amino acid replacements per position. Neighbor-joining (NJ) trees were obtained with NEIGHBOR and the most parsimonious trees were determined with PROTPARS. Robustness of these trees was evaluated by maximum likelihood (ML) analysis with PHYML [37] and Bayesian estimation of phylogeny with MrBayes [38].

### Metabolic labeling of *E. faecalis *with ^75^Se and purification of Se-binding proteins

To examine for the occurrence of Se-binding proteins in *E. faecalis*, 50 ml of *E. faecalis *strain 29212 (ATCC) in BHI media (Gibco) and *E. coli *strain Nova Blue (Novagen) in LB media (MP Bio) were metabolically labeled with 50 μCi of ^75^Se ([^75^Se]selenious acid (specific activity, 1,000 Ci/mmol) Research Reactor Facility, University of Missouri (Columbia, Mo.)) for 24 h. Bacterial cultures were grown at 37°C in the dark with extensive agitation (250 rpm) to provide sufficient aeration. For anaerobic growth (preferred for *E. faecalis*), the cultures were cultivated in fully filled, parafilm-sealed tubes without shaking. Cells were collected, resuspended in PBS buffer and sonicated. 30 μg of total soluble protein from each organism were resolved by 10% native or SDS-PAGE under non-reducing conditions and transferred onto a PVDF membrane (Invitrogen). ^75^Se-labeled proteins were visualized with a PhosphorImager. To enrich for Se-binding proteins, 50 ml of *E. faecalis *cells were labeled with ^75^Se for 24 h at 37°C without shaking in low oxygen conditions and the labeled cells were mixed with 5 g of unlabeled cells that were cultured separately using the same procedure. Cells were washed twice in cold PBS, resuspended in PBS containing EDTA-free protease inhibitor mixture (Roche), and Se-binding proteins were fractionated under non-reducing conditions on DEAE-Sepharose and Phenyl-Sepharose (GE Healthcare) columns following ^75^Se radioactivity in protein fractions. Fractions containing ^75^Se were collected and analyzed by SDS-PAGE.

### Analysis of *E. faecalis *Se-binding proteins

Chromatographically enriched Se-binding proteins from *E. faecalis *were subjected heat and reducing agent treatments. Protein samples (~30 μg of total protein) were treated by heating (90°C for 10 min) in SDS sample buffer (Invitrogen) with or without 10 mM DTT or 2-mercaptoethanol, and then subjected to SDS-PAGE.

## Notes

During the review of this manuscript, another study was published (Haft DH and Self WT. *Biol Direct*. 2008. 3: 4) which proposed HP1 (EF2563) and selD as markers for the identification of selenium-dependent molybdenum hydroxylases [39]. These data are consistent with the idea that a new SelD-associated Se utilization pathway is present in certain organisms such as *E. faecalis*.

## Authors' contributions

YZ and VNG designed the study. YZ carried out computational studies, including comparative genomics, sequence alignment, phylogenetic analysis and drafted the manuscript. AAT carried out the experimental studies, including ^75^Se labeling, protein purification and SDS-PAGE analysis, and helped to draft the manuscript. DLH and VNG analyzed the data and edited the manuscript. All authors read and approved the final manuscript.

## Supplementary Material

Additional file 1 This file includes table S1 which shows the occurrance of Sec and SeU utilization traits in each sequenced organism. Click here for file

Additional file 2 This file includes supplemental figure S1 which shows genomic context of selD in additional orphan SelD-containing genomes. Click here for file
