# Supplementary material for: In silico identification of genes involved in selenium metabolism: evidence for a third selenium utilization trait
Source: BMC Genomics. 2008 May 29;9:251. doi: 10.1186/1471-2164-9-251 (PMC2432076; doi:10.1186/1471-2164-9-251)

Supplemental figures

Figure legends

Figure S1 - Genomic context of SelD in orphan SelD-containing genomes

Candidate genes involved in the new selenium utilization trait are color coded. Coding direction is also indicated.

Figure S1

Anaerostipes caccae

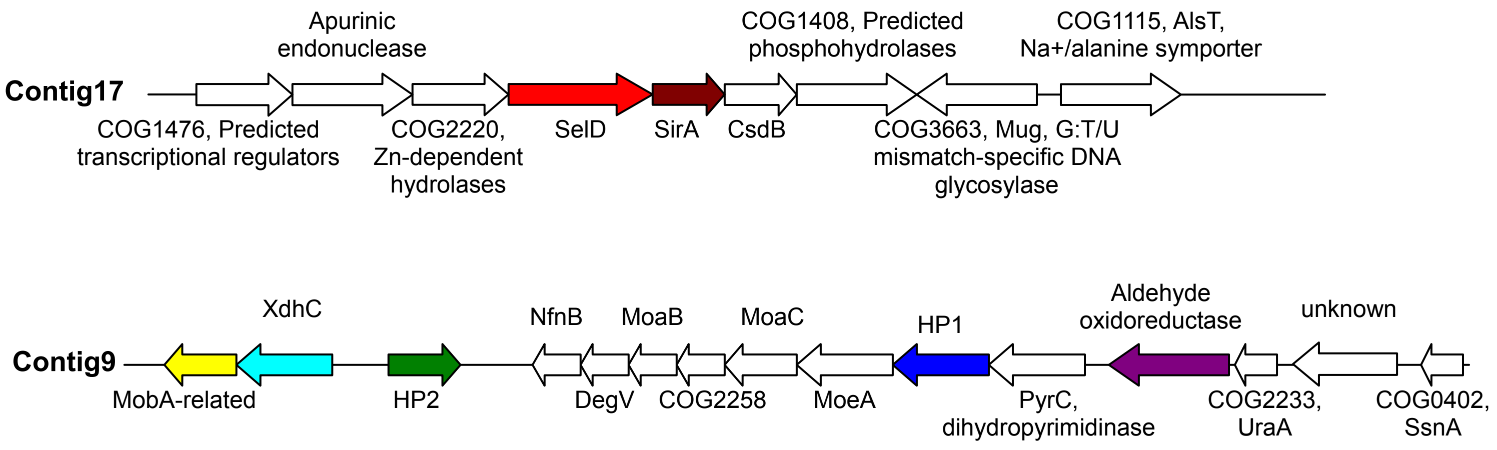

Ruminococcus obeum

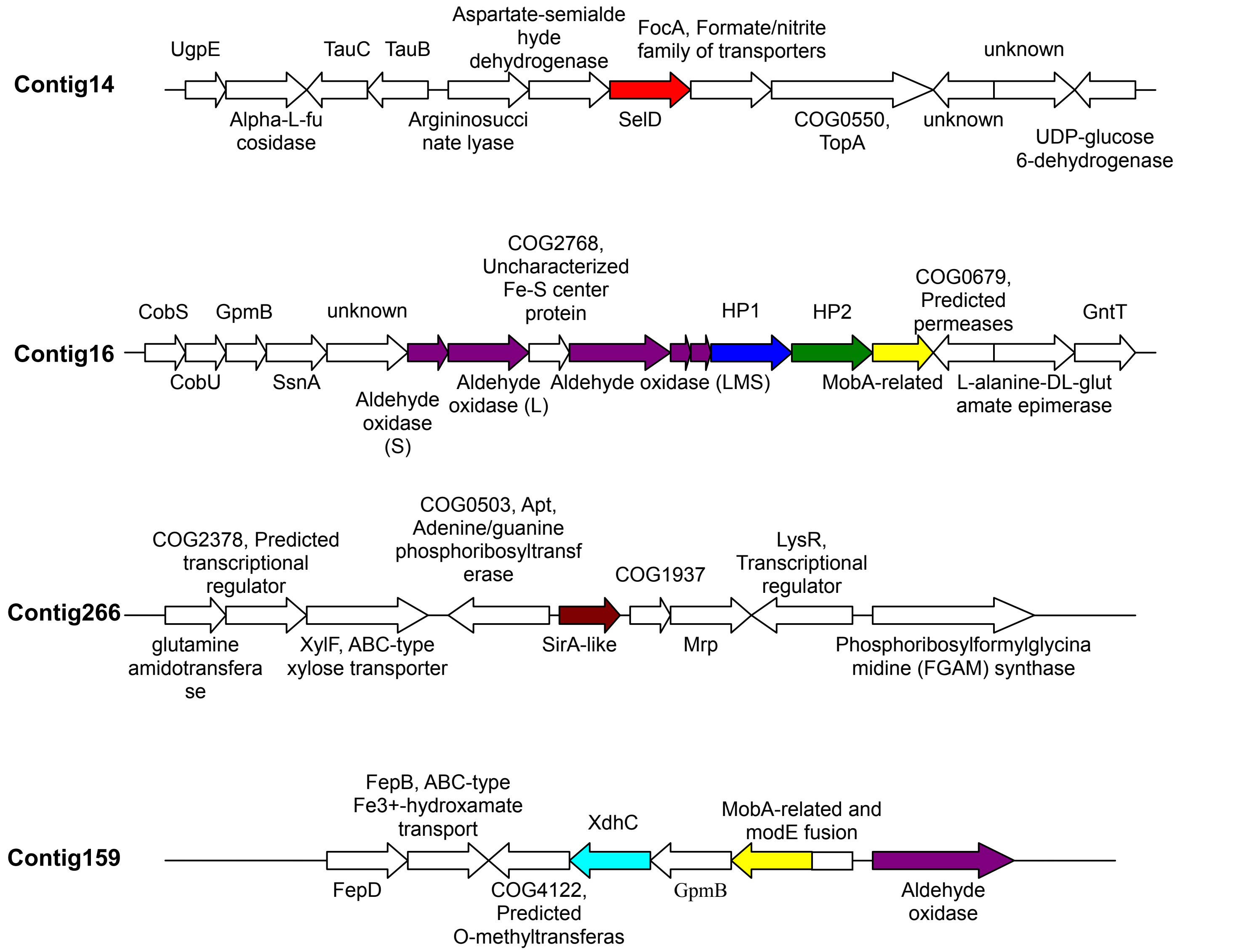

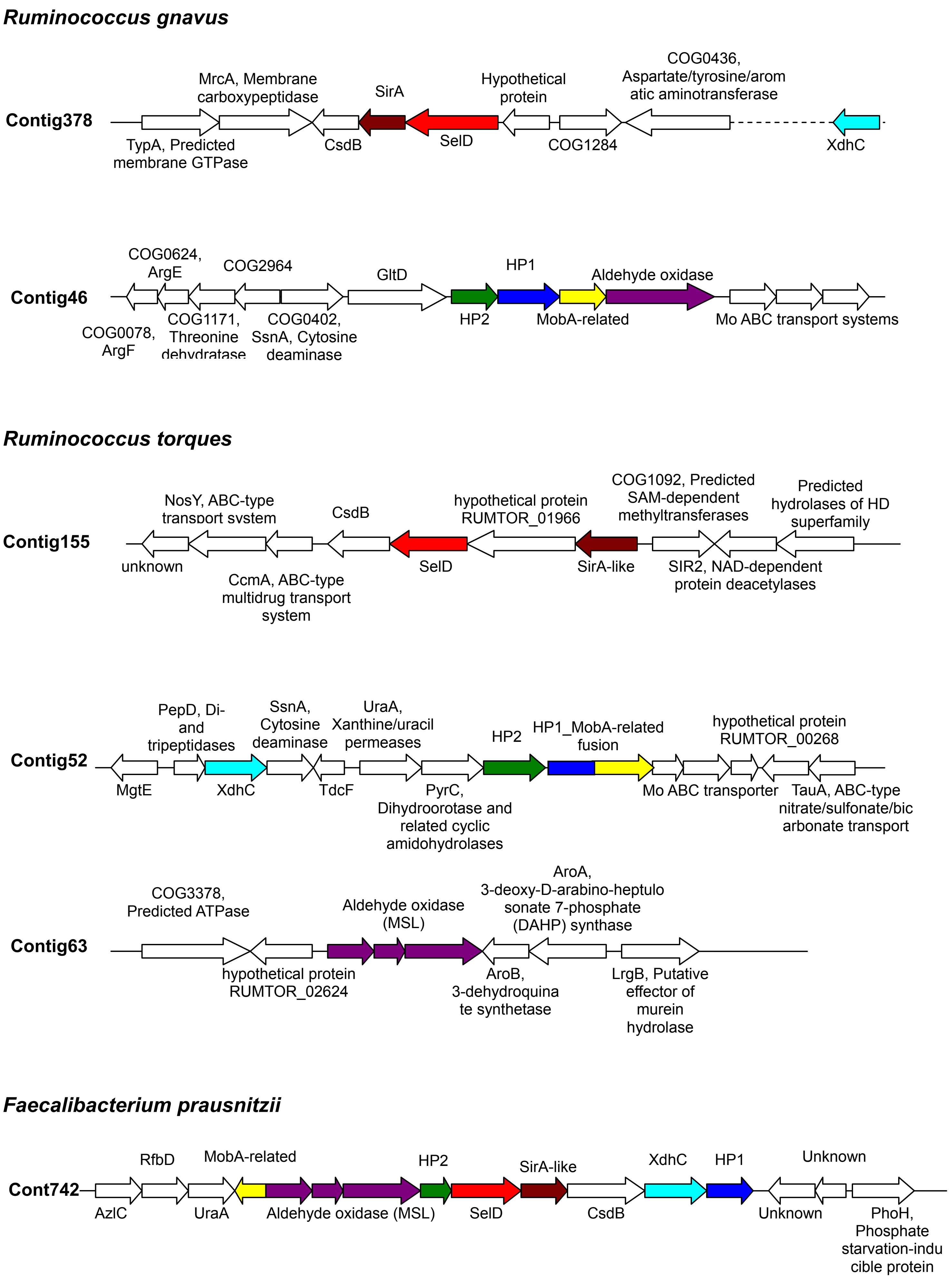
Ruminococcus gnavus
Contig378
MrcA, Membrane carboxypeptidase
SirA
Hypothetical protein
COG0436, Aspartate/tyrosine/aromatic aminotransferase
TypA, Predicted membrane GTPase
CsdB
SelD
COG1284
XdhC
Contig46
COG0624, ArgE
COG2964
GltD
HP1
Aldehyde oxidase
COG0078, ArgF
COG1171, Threonine dehydratase
COG0402, SsnA, Cytosine deaminase
HP2
MobA-related
Mo ABC transport systems
Ruminococcus torques
Contig155
NosY, ABC-type transport system
CsdB
hypothetical protein RUMTOR_01966
COG1092, Predicted SAM-dependent methyltransferases
Predicted hydrolases of HD superfamily
unknown
CcmA, ABC-type multidrug transport system
SelD
SirA-like
SIR2, NAD-dependent protein deacetylases
Contig52
PepD, Di- and tripeptidases
SsnA, Cytosine deaminase
UraA, Xanthine/uracil permeases
HP2
HP1_MobA-related fusion
hypothetical protein RUMTOR_00268
MgtE
XdhC
TdcF
PyrC, Dihydroorotase and related cyclic amidohydrolases
Mo ABC transporter
TauA, ABC-type nitrate/sulfonate/bicarbonate transport
Contig63
COG3378, Predicted ATPase
Aldehyde oxidase (MSL)
AroA, 3-deoxy-D-arabino-heptulosonate 7-phosphate (DAHP) synthase
hypothetical protein RUMTOR_02624
AroB, 3-dehydroquinate synthetase
LrgB, Putative effector of murein hydrolase
Faecalibacterium prausnitzii
Cont742
RfbD
MobA-related
HP2
SirA-like
XdhC
HP1
Unknown
AzlC
UraA
Aldehyde oxidase (MSL)
SelD
CsdB
Unknown
PhoH, Phosphate starvation-inducible protein

Vibrio shilonii

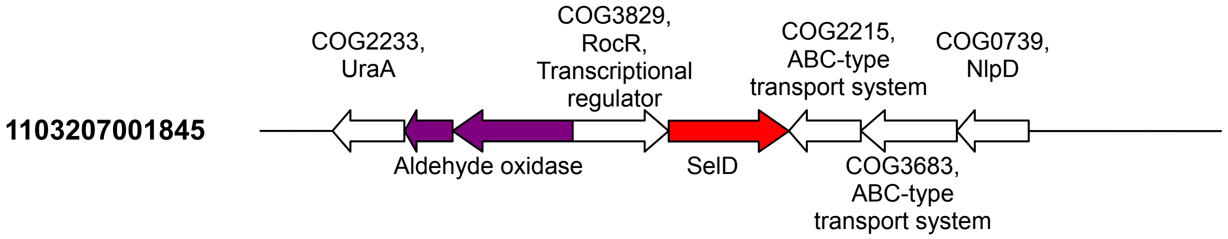

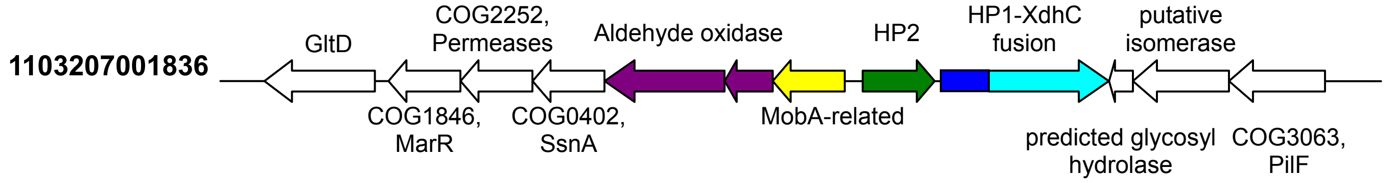

Clostridium butyricum

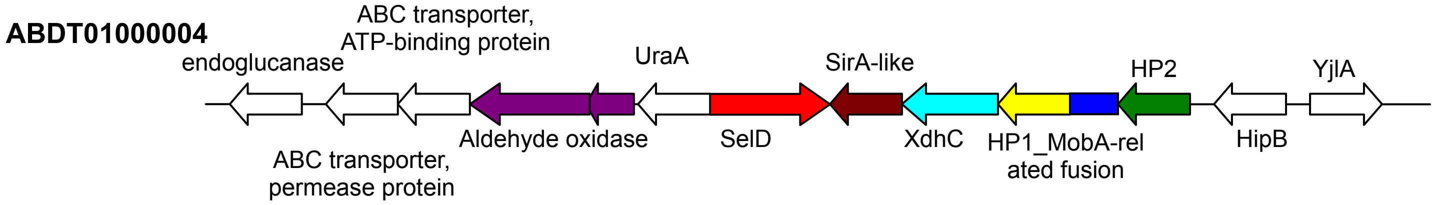

Supplement: Additional file 1 — This file includes table S1 which shows the occurrance of Sec and SeU utilization traits in each sequenced organism. [file 1471-2164-9-251-S1.pdf]
