# Supplementary material for: In silico identification of genes involved in selenium metabolism: evidence for a third selenium utilization trait
Source: BMC Genomics. 2008 May 29;9:251. doi: 10.1186/1471-2164-9-251 (PMC2432076; doi:10.1186/1471-2164-9-251)
Supplement: Additional file 2 — This file includes supplemental figure S1 which shows genomic context of selD in additional orphan SelD-containing genomes. [file 1471-2164-9-251-S2.pdf]

Table S1. Distribution of SeC and SeU utilization trait in prokaryotes

Classification	Organism	SelD	Sec trait	Selenouridine trait
**Bacteria**				
**Actinobacteria**	Acidothermus cellulolyticus	-	-	-
	Actinomyces odontolyticus	-	-	-
	Arthrobacter aurescens	-	-	-
	Arthrobacter sp. FB24	-	-	-
	Bifidobacterium adolescentis	-	-	-
	Bifidobacterium longum	-	-	-
	Brevibacterium linens	+C	+	-
	Clavibacter michiganensis	-	-	-
	Collinsella aerofaciens	+U	+	-
	Corynebacterium diphtheriae	-	-	-
	Corynebacterium efficiens	-	-	-
	Corynebacterium glutamicum	-	-	-
	Corynebacterium jeikeium	-	-	-
	Frankia alni	-	-	-
	Frankia sp. CcI3	-	-	-
	Janibacter sp.	-	-	-
	Kineococcus radiotolerans	+C	+	-
	Leifsonia xyli	-	-	-
	Mycobacterium avium	+C	+	-
	Mycobacterium bovis	-	-	-
	Mycobacterium flavescens	-	-	-
	Mycobacterium gilvum	-	-	-
	Mycobacterium leprae	+C	+	-
	Mycobacterium smegmatis	+C	+	-
	Mycobacterium sp.	+C	+	-
	Mycobacterium tuberculosis	-	-	-
	Mycobacterium ulcerans	-	-	-
	Mycobacterium vanbaalenii	-	-	-
	Nocardia farcinica	-	-	-
	Nocardioides sp.	-	-	-
	Propionibacterium acnes	-	-	-
	Rhodococcus sp.	-	-	-
	Rubrobacter xylanophilus	+U	+	-
	Saccharopolyspora erythraea	+C	+	-
	Salinispora arenicola	-	-	-

	Salinispora tropica	+C	+	-
	Streptomyces avermitilis	-	-	-
	Streptomyces coelicolor	-	-	-
	Thermobifida fusca	-	-	-
	Tropheryma whipplei	-	-	-
	marine actinobacterium PHSC20C1	-	-	-
**Bacteroidetes**	Algoriphagus sp.	+C	-	+
	Bacteroides caccae	-	-	-
	Bacteroides capillosus	+C	+	-
	Bacteroides fragilis	-	-	-
	Bacteroides ovatus	-	-	-
	Bacteroides thetaiotaomicron	-	-	-
	Bacteroides uniformis	-	-	-
	Bacteroides vulgatus	-	-	-
	Candidatus Sulcia muelleri	-	-	-
	Cellulophaga sp.	-	-	-
	Croceibacter atlanticus	-	-	-
	Cytophaga hutchinsonii	-	-	-
	Dokdonia donghaensis	-	-	-
	Flavobacteria bacterium	-	-	-
	Flavobacteriales bacterium	-	-	-
	Flavobacterium johnsoniae	-	-	-
	Flavobacterium psychrophilum	-	-	-
	Gramella forsetii	-	-	-
	Leeuwenhoekiella blandensis	-	-	-
	Microscilla marina	-	-	-
	Parabacteroides distasonis	-	-	-
	Parabacteroides merdae	-	-	-
	Pedobacter sp. BAL39	-	-	-
	Polaribacter dokdonensis	+C	-	+
	Polaribacter irgensii	+C	-	+
	Porphyromonas gingivalis	+C	-	+
	Psychroflexus torquis	-	-	-
	Robiginitalea biformata	-	-	-
	Salinibacter ruber	-	-	-
	Tenacibaculum sp.	-	-	-
	unidentified eubacterium SCB49	-	-	-
Chlorobi	Chlorobium chlorochromatii	-	-	-
	Chlorobium ferrooxidans	-	-	-
	Chlorobium limicola	-	-	-

	Chlorobium phaeobacteroides	-	-	-
	Chlorobium tepidum	-	-	-
	Pelodictyon luteolum	-	-	-
	Pelodictyon phaeoclathratiforme	-	-	-
	Prosthecochloris aestuarii	-	-	-
	Prosthecochloris vibrioformis	-	-	-
**Chlamydiae**	Candidatus Protochlamydia amoebophila	-	-	-
	Chlamydia muridarum	-	-	-
	Chlamydia trachomatis	-	-	-
	Chlamydophila abortus	-	-	-
	Chlamydophila caviae	-	-	-
	Chlamydophila felis	-	-	-
	Chlamydophila pneumoniae	-	-	-
**Cyanobacteria**	Acaryochloris marina	+C	-	+
	Anabaena variabilis	-	-	-
	Crocosphaera watsonii	+C	-	+
	Cyanothece sp.	+C	-	+
	Gloeobacter violaceus	-	-	-
	Leptolyngbya valderiana	-	-	-
	Lyngbya sp.	+C	-	+
	Nodularia spumigena	-	-	-
	Nostoc punctiforme	-	-	-
	Nostoc sp.	-	-	-
	Prochlorococcus marinus	+C	-	+
	Synechococcus elongatus	-	-	-
	Synechococcus sp.	+C	-	+
	Synechocystis sp.	-	-	-
	Thermosynechococcus elongatus	-	-	-
	Trichodesmium erythraeum	+C	-	+
**Firmicutes/Bacillales**	Bacillus amyloliquefaciens	-	-	-
	Bacillus anthracis	-	-	-
	Bacillus cereus	-	-	-
	Bacillus clausii	-	-	-
	Bacillus coagulans	+C	-	+
	Bacillus halodurans	-	-	-
	Bacillus licheniformis	-	-	-
	Bacillus pumilus	-	-	-
	Bacillus sp. NRRL	+U	+	+
	Bacillus subtilis	-	-	-

	Bacillus thuringiensis	-	-	-
	Bacillus weihenstephanensis	-	-	-
	Exiguobacterium sibiricum	-	-	-
	Geobacillus kaustophilus	-	-	-
	Geobacillus thermodenitrificans	-	-	-
	Listeria innocua	-	-	-
	Listeria monocytogenes	-	-	-
	Listeria welshimeri	-	-	-
	Oceanobacillus iheyensis	-	-	-
	Paenibacillus larvae	+C	-	+
	Pasteuria nishizawae	-	-	-
	Staphylococcus aureus	-	-	-
	Staphylococcus epidermidis	-	-	-
	Staphylococcus haemolyticus	-	-	-
	Staphylococcus saprophyticus	-	-	-
**Firmicutes/Clostridia**	Alkaliphilus metalliredigens	+C	+	+
	Alkaliphilus oremlandii	+U	+	+
	Anaerostipes caccae	+C	-	-
	Caldicellulosiruptor saccharolyticus	-	-	-
	Carboxydothermus hydrogenoformans	+U	+	+
	Clostridium acetobutylicum	-	-	-
	Clostridium beijerincki	-	-	-
	Clostridium bolteae	+U	+	-
	Clostridium botulinum	+U	+	-
	Clostridium butyricum	+C	-	-
	Clostridium cellulolyticum	-	-	-
	Clostridium difficile	+U	+	+
	Clostridium kluyveri	-	-	-
	Clostridium leptum	-	-	-
	Clostridium novyi	-	-	-
	Clostridium perfringens	+U	+	+
	Clostridium phytofermentans	+C	-	-
	Clostridium sp. OhILAs	+U	+	+
	Clostridium tetani	-	-	-
	Clostridium thermocellum	-	-	-
	Desulfitobacterium hafniense	+C	+	-
	Desulfotomaculum reducens	+U	+	+
	Dorea longicatena	+U	+	-

	Epulopiscium sp.	+C	-	+
	Eubacterium siraeum	-	-	-
	Eubacterium ventriosum	-	-	-
	Faecalibacterium prausnitzii	+C	-	-
	Halothermothrix orenii	-	-	-
	Moorella thermoacetica	+U	+	-
	Pelotomaculum thermopropionicum	+U	+	+
	Peptostreptococcus micros	+U	+	-
	Ruminococcus gnavus	+C	-	-
	Ruminococcus obeum	+C	-	-
	Ruminococcus torques	+C	-	-
	Syntrophomonas wolfei	+U	+	-
	Thermoanaerobacter ethanolicus	-	-	-
	Thermoanaerobacter tengcongensis	+U	+	+
	Thermosinus carboxydivorans	+U	+	+
**Firmicutes/Lactobacillales**	Enterococcus faecalis	+C	-	-
	Enterococcus faecium	-	-	-
	Lactobacillus acidophilus	-	-	-
	Lactobacillus brevis	-	-	-
	Lactobacillus casei	-	-	-
	Lactobacillus delbrueckii	-	-	-
	Lactobacillus gasseri	-	-	-
	Lactobacillus johnsonii	-	-	-
	Lactobacillus plantarum	-	-	-
	Lactobacillus reuteri	-	-	-
	Lactobacillus sakei	-	-	-
	Lactobacillus salivarius	-	-	-
	Lactococcus lactis	-	-	-
	Leuconostoc mesenteroides	-	-	-
	Oenococcus oeni	-	-	-
	Pediococcus pentosaceus	-	-	-
	Streptococcus agalactiae	-	-	-
	Streptococcus gordonii	-	-	-
	Streptococcus mutans	-	-	-
	Streptococcus pneumoniae	-	-	-
	Streptococcus pyogenes	-	-	-
	Streptococcus sanguinis	-	-	-
	Streptococcus suis	-	-	-
	Streptococcus thermophilus	-	-	-

	Symbiobacterium thermophilum	+U	+	-
**Firmicutes/Mollicutes**	Aster yellows witches'-broom phytoplasma	-	-	-
	Eubacterium dolichum	-	-	-
	Mesoplasma florum	-	-	-
	Mycoplasma agalactiae	-	-	-
	Mycoplasma capricolum	-	-	-
	Mycoplasma gallisepticum	-	-	-
	Mycoplasma genitalium	-	-	-
	Mycoplasma hyopneumoniae	-	-	-
	Mycoplasma mobile	-	-	-
	Mycoplasma mycoides	-	-	-
	Mycoplasma penetrans	-	-	-
	Mycoplasma pneumoniae	-	-	-
	Mycoplasma pulmonis	-	-	-
	Mycoplasma synoviae	-	-	-
	Onion yellows phytoplasma	-	-	-
	Ureaplasma parvum	-	-	-
	Ureaplasma urealyticum	-	-	-
**Acidobacteria**	Acidobacteria bacterium Ellin345	+U	+	-
	Solibacter usitatus	+U	+	-
**Aquificae**	Aquifex aeolicus	+U	+	-
	Hydrogenobaculum sp. Y04AAS1	-	-	-
Planctomycetes	Blastopirellula marina	-	-	-
	Rhodopirellula baltica	-	-	-
	Planctomyces maris	-	-	-
**Chloroflexi**	Chloroflexus aggregans	-	-	-
	Chloroflexus aurantiacus	+U	+	-
	Dehalococcoides ethenogenes	-	-	-
	Dehalococcoides sp.	-	-	-
	Roseiflexus sp.	-	-	-
	Herpetosiphon aurantiacus	+U	+	-
	Roseiflexus castenholzii	-	-	-
Deinococcus-Thermus	Deinococcus geothermalis	-	-	-
	Deinococcus radiodurans	-	-	-
	Thermus thermophilus	-	-	-
Fusobacteria	Fusobacterium nucleatum	-	-	-
**Thermotogae**	Thermosipho melanesiensis	-	-	-
	Thermotoga lettingae	-	-	-
	Thermotoga maritima	-	-	-

	Thermotoga petrophila	-	-	-
	Fervidobacterium nodosum	-	-	-
	Petrotoga mobilis	-	-	-
**Lentisphaerae**	Lentisphaera araneosa	-	-	-
	Victivallis vadensis	-	-	-
**candidate division TM7**	candidate division TM7 isolate TM7a	-	-	-
	candidate division TM7 isolate TM7b	-	-	-
	candidate division TM7 isolate TM7c	-	-	-
**Verrucomicrobia**	Opitutaceae bacterium TAV2	-	-	-
**Proteobacteria/others**	Magnetococcus sp.	+C	-	+
	Mariprofundus ferrooxydans (Siderooxidans marinum)	-	-	-
**Proteobacteria/alpha/others**	Acidiphilium cryptum	-	-	-
	Aurantimonas sp.	-	-	-
	Azorhizobium caulinodans	+C	+	-
	Bartonella bacilliformis	-	-	-
	Bartonella henselae	-	-	-
	Bartonella quintana	-	-	-
	Bradyrhizobium japonicum	-	-	-
	Bradyrhizobium sp.	-	-	-
	Brucella abortus	-	-	-
	Brucella melitensis	-	-	-
	Brucella ovis	-	-	-
	Brucella suis	-	-	-
	Caulobacter crescentus	-	-	-
	Caulobacter sp.	-	-	-
	Dinoroseobacter shibae	+C	-	+
	Erythrobacter litoralis	-	-	-
	Erythrobacter sp.	-	-	-
	Fulvimarina pelagi	-	-	-
	Gluconobacter oxydans	-	-	-
	Granulibacter bethesdensis	-	-	-
	Hyphomonas neptunium	+C	+	+
	Jannaschia sp.	+C	-	+
	Labrenzia aggregata	-	-	-
	Loktanella vestfoldensis	+C	-	+
	Magnetospirillum magneticum	-	-	-
	Magnetospirillum magnetotacticum	-	-	-

	Maricaulis maris	+C	-	+
	Mesorhizobium loti	-	-	-
	Mesorhizobium sp.	-	-	-
	Methylobacterium chloromethanicum	-	-	-
	Methylobacterium extorquens	-	-	-
	Methylobacterium sp.	+C	+	-
	Nitrobacter hamburgensis	-	-	-
	Nitrobacter sp.	-	-	-
	Nitrobacter winogradskyi	-	-	-
	Novosphingobium aromaticivorans	-	-	-
	Oceanicaulis alexandrii	+C	-	+
	Oceanicola batsensis	+C	-	+
	Oceanicola granulosus	+C	-	+
	Ochrobactrum anthropi	+C	+	-
	Paracoccus denitrificans	+C	+	+
	Parvibaculum lavamentivorans	-	-	-
	Parvularcula bermudensis	-	-	-
	Rhodobacter sphaeroides	+C	-	+
	Rhodobacterales bacterium	+C	-	+
	Rhodopseudomonas palustris	-	-	-
	Rhodospirillum rubrum	-	-	-
	Roseobacter denitrificans	-	-	-
	Roseobacter sp.	+C	-	+
	Roseovarius nubinhibens	-	-	-
	Roseovarius sp.	+C	-	+
	Sagittula stellata	+C	-	+
	Silicibacter pomeroyi	+C	-	+
	Silicibacter sp.	+C	-	+
	Sphingomonas sp.	-	-	-
	Sphingomonas wittichii	-	-	-
	Sphingopyxis alaskensis	-	-	-
	Stappia aggregata	-	-	-
	Sulfitobacter sp.	+C	+	-
	Xanthobacter autotrophicus	+C	+	-
	Zymomonas mobilis	-	-	-
	alpha proteobacterium HTCC2255	-	-	-
**Proteobacteria/alpha/Rhizobiaceae**	Agrobacterium tumefaciens	-	-	-
	Rhizobium etli	-	-	-

	Rhizobium leguminosarum	-	-	-
	Sinorhizobium medicae	-	-	-
	Sinorhizobium meliloti	+C	+	-
**Proteobacteria/alpha/Rickettsiales**	Anaplasma marginale	-	-	-
	Anaplasma phagocytophilum	-	-	-
	Candidatus Pelagibacter ubique	-	-	-
	Ehrlichia canis	-	-	-
	Ehrlichia chaffeensis	-	-	-
	Ehrlichia ruminantium	-	-	-
	Neorickettsia sennetsu	-	-	-
	Orientia tsutsugamushi	-	-	-
	Rickettsia africae	-	-	-
	Rickettsia akari	-	-	-
	Rickettsia bellii	-	-	-
	Rickettsia canadensis	-	-	-
	Rickettsia conorii	-	-	-
	Rickettsia felis	-	-	-
	Rickettsia massiliae	-	-	-
	Rickettsia prowazekii	-	-	-
	Rickettsia rickettsii	-	-	-
	Rickettsia sibirica	-	-	-
	Rickettsia typhi	-	-	-
	Wolbachia endosymbiont	-	-	-
**Proteobacteria/beta/Bordetella**	Bordetella bronchiseptica	+C	-	+
	Bordetella parapertussis	-	-	-
	Bordetella pertussis	-	-	-
**Proteobacteria/beta/Burkholderiaceae**	Burkholderia ambifaria	+C	+	+
	Burkholderia cenocepacia	+C	+	+
	Burkholderia dolosa	+C	+	+
	Burkholderia mallei	+C	+	-
	Burkholderia multivorans	+C	+	+
	Burkholderia oklahomensis	+C	+	+
	Burkholderia phymatum	+C	+	+
	Burkholderia phytofirmans	+C	+	+
	Burkholderia pseudomallei	+C	+	+
	Burkholderia sp. 383	+C	+	+
	Burkholderia thailandensis	+C	+	+

	Burkholderia ubonensis	+C	+	+
	Burkholderia vietnamiensis	+C	+	+
	Burkholderia xenovorans	+C	+	+
	Limnobacter sp.	+C	-	+
	Polynucleobacter sp.	+C	-	+
	Ralstonia eutropha	+C	-	+
	Ralstonia metallidurans	+C	-	+
	Ralstonia pickettii	-	-	-
	Ralstonia solanacearum	-	-	-
**Proteobacteria/beta/Neisseriaceae**	Chromobacterium violaceum	-	-	-
	Neisseria gonorrhoeae	-	-	-
	Neisseria meningitidis	-	-	-
**Proteobacteria/beta/others**	Acidovorax avenae	-	-	-
	Acidovorax sp.	+C	-	+
	Azoarcus sp.	+C	-	+
	Comamonas testosteroni	+C	+	+
	Dechloromonas aromatica	+C	+	+
	Delftia acidovorans	+C	+	+
	Herminiimonas arsenicoxydans	+C	-	+
	Janthinobacterium sp.	+C	-	+
	Methylibium petroleiphilum	+C	-	+
	Methylobacillus flagellatus	-	-	-
	Methylophilales bacterium	-	-	-
	Nitrosomonas europaea	+C	-	+
	Nitrosomonas eutropha	+C	-	+
	Nitrosospira multiformis	-	-	-
	Polaromonas naphthalenivorans	+C	-	+
	Polaromonas sp.	+C	-	+
	Rhodoferax ferrireducens	+C	-	+
	Thiobacillus denitrificans	+C	-	+
	Verminephrobacter eiseniae	+C	-	+
**Proteobacteria/delta**	Anaeromyxobacter dehalogenans	+U	+	-
	Anaeromyxobacter sp.	+U	+	-
	Bdellovibrio bacteriovorus	+C	-	+
	Candidatus Desulfococcus oleovorans	+U	+	-
	Desulfotalea psychrophila	+U	+	+
	Desulfovibrio desulfuricans	+U	+	-
	Desulfovibrio vulgaris	+U	+	+

	Desulfuromonas acetoxidans	+U	+	+
	Geobacter bemidjiensis	+U	+	+
	Geobacter lovleyi	+U	+	+
	Geobacter metallireducens	+U	+	+
	Geobacter sp. FRC-32	**+U**	**+**	**+**
	Geobacter sulfurreducens	+U	+	+
	Geobacter uraniumreducens	+U	+	+
	Lawsonia intracellularis	-	+	-
	Myxococcus xanthus	+C	+	-
	Pelobacter carbinolicus	+U	+	+
	Pelobacter propionicus	+C	-	+
	Plesiocystis pacifica	+C	+	-
	Stigmatella aurantiaca	+C	+	-
	Syntrophobacter fumaroxidans	+U	+	-
	Syntrophus aciditrophicus	+U	+	-
	delta proteobacterium MLMS-1	+U	+	-
**Proteobacteria/epsilon**	Arcobacter butzleri	+C	+	-
	Caminibacter mediatlanticus	+U	+	-
	Campylobacter coli	+U	+	+
	Campylobacter concisus	+U	+	+
	Campylobacter curvus	+U	+	+
	Campylobacter fetus	+U	+	-
	Campylobacter hominis	+U	+	-
	Campylobacter jejuni	+U	+	+
	Campylobacter lari	+U	+	+
	Campylobacter upsaliensis	+U	+	+
	Helicobacter acinonychis	-	-	-
	Helicobacter hepaticus	+C	+	-
	Helicobacter pylori	-	-	-
	Nitratiruptor sp.	-	-	-
	Sulfurovum sp.	+C	-	+
	Thiomicrospira denitrificans	+U	+	+
	Wolinella succinogenes	+C	+	+
**Proteobacteria/gamma/Enterobacteriales**	Buchnera aphidicola	-	-	-
	Candidatus Blochmannia	-	-	-
	Citrobacter koseri	+C	+	+
	Enterobacter sakazakii	+C	+	+
	Enterobacter sp.	+C	+	+

	Erwinia carotovora	-	-	-
	Escherichia coli	+C	+	+
	Klebsiella pneumoniae	+C	+	+
	Photorhabdus luminescens	+C	+	-
	Salmonella enterica	+C	+	+
	Salmonella typhimurium	+C	+	+
	Serratia proteamaculans	+C	+	+
	Shigella boydii	+C	+	+
	Shigella dysenteriae	+C	+	+
	Shigella flexneri 2a	+C	+	+
	Shigella sonnei	+C	+	+
	Sodalis glossinidius	-	-	-
	Wigglesworthia glossinidia	-	-	-
	Yersinia bercovieri	+C	+	-
	Yersinia enterocolitica	+C	+	-
	Yersinia frederiksenii	+C	+	-
	Yersinia intermedia	+C	+	-
	Yersinia mollaretii	+C	+	-
	Yersinia pestis KIM	+C	+	-
	Yersinia pseudotuberculosis	+C	+	-
**Proteobacteria/gamma/Others**	Acinetobacter baumannii	-	-	-
	Acinetobacter sp.	-	-	-
	Aeromonas hydrophila	+C	+	+
	Aeromonas salmonicida	+C	+	+
	Alcanivorax borkumensis	+C	-	+
	Alkalilimnicola ehrlichei	-	-	-
	Alteromonadales bacterium	-	-	-
	Alteromonas macleodii	-	-	-
	Baumannia cicadellinicola	-	-	-
	Beggiatoa sp.	-	-	-
	Candidatus Carsonella	-	-	-
	Candidatus Ruthia magnifica	-	-	-
	Candidatus Vesicomyosocius	-	-	-
	Chromohalobacter salexigens	+C	-	+
	Colwellia psychrerythraea	+C	-	+
	gamma proteobacterium (Congregibacter litoralis) KT 71	+C	-	+
	Coxiella burnetii	-	-	-

	Dichelobacter nodosus	-	-	-
	Endoriftia persephone	-	-	-
	Francisella tularensis	-	-	-
	Hahella chejuensis	+C	-	+
	Halorhodospira halophila	-	-	-
	Idiomarina baltica	-	-	-
	Idiomarina loihiensis	-	-	-
	Legionella pneumophila	-	-	-
	Marinobacter algicola	+C	-	+
	Marinobacter aquaeolei	+C	-	+
	Marinobacter sp.	+C	-	+
	Marinomonas sp.	-	-	-
	Methylococcus capsulatus	-	-	-
	Moritella sp.	-	-	-
	Neptuniibacter caesariensis	-	-	-
	Nitrococcus mobilis	-	-	-
	Nitrosococcus oceani	-	-	-
	Oceanobacter sp.	-	-	-
	Pseudoalteromonas atlantica	-	-	-
	Pseudoalteromonas haloplanktis	-	-	-
	Pseudoalteromonas tunicata	-	-	-
	Psychrobacter arcticus	-	-	-
	Psychrobacter cryohalolentis	-	-	-
	Psychrobacter sp.	-	-	-
	Psychromonas ingrahamii	+C	-	+
	Psychromonas sp.	+C	+	+
	Reinekea sp.	+C	-	+
	Rickettsiella grylli	-	-	-
	Saccharophagus degradans	-	-	-
	Shewanella amazonensis	+C	-	+
	Shewanella baltica	+C	+	+
	Shewanella denitrificans	+C	-	+
	Shewanella frigidimarina	+C	-	+
	Shewanella loihica	+C	-	+
	Shewanella oneidensis	+C	+	+
	Shewanella pealeana	+C	+	+
	Shewanella putrefaciens	+C	-	+
	Shewanella sediminis	+C	-	+
	Shewanella sp.	+C	+	+
	Shewanella woodyi	+C	-	+

	Thiomicrospira crunogena	+C	-	+
	Vibrionales bacterium	-	-	-
	marine gamma proteobacterium HTCC2080	+C	+	+
	marine gamma proteobacterium HTCC2143	+C	+	+
	marine gamma proteobacterium HTCC2207	+C	-	+
**Proteobacteria/gamma/Pasteurellaceae**	Actinobacillus pleuropneumoniae	+U	+	-
	Actinobacillus succinogenes	+U	+	-
	Haemophilus ducreyi	+U	+	-
	Haemophilus influenzae	+U	+	-
	Haemophilus somnus	-	-	-
	Mannheimia haemolytica	+U	+	-
	Mannheimia succiniciproducens	+U	+	-
	Pasteurella multocida	+C	+	-
**Proteobacteria/gamma/Pseudomonadaceae**	Azotobacter vinelandii	+C	-	+
	Pseudomonas aeruginosa	+C	+	+
	Pseudomonas entomophila	+C	+	+
	Pseudomonas fluorescens PfO-1	+C	+	+
	Pseudomonas mendocina	+C	-	+
	Pseudomonas putida	+C	+	+
	Pseudomonas stutzeri	+C	+	+
	Pseudomonas syringae	+C	-	+
**Proteobacteria/gamma/Vibrionaceae**	Photobacterium profundum	+U	+	+
	Photobacterium sp.	+C	+	+
	Vibrio alginolyticus	-	-	-
	Vibrio angustum	+C	+	+
	Vibrio cholerae	-	-	-
	Vibrio fischeri	-	-	-
	Vibrio harveyi	-	-	-
	Vibrio parahaemolyticus	-	-	-
	Vibrio shilonii	+C	-	-
	Vibrio sp.	-	-	-
	Vibrio splendidus	-	-	-
	Vibrio vulnificus	-	-	-
**Proteobacteria/gamma/Xanthomonadaceae**	Stenotrophomonas maltophilia	+C	+	-
	Xanthomonas axonopodis	-	-	-

	Xanthomonas campestris	-	-	-
	Xanthomonas oryzae	-	-	-
	Xylella fastidiosa	-	-	-
**Spirochaetales**	Borrelia afzelii	-	-	-
	Borrelia burgdorferi	-	-	-
	Borrelia garinii	-	-	-
	Borrelia valaisiana	-	-	-
	Leptospira borgpetersenii	-	-	-
	Leptospira interrogans	-	-	-
	Treponema denticola	+U	+	-
	Treponema pallidum	-	-	-
**Archaea**				
**Crenarchaeota/Desulfurococcales**	Aeropyrum pernix	-	-	-
	Hyperthermus butylicus	-	-	-
	Ignicoccus hospitalis	-	-	-
	Staphylothermus marinus	-	-	-
**Crenarchaeota/Sulfolobales**	Metallosphaera sedula	-	-	-
	Sulfolobus acidocaldarius	-	-	-
	Sulfolobus solfataricus	-	-	-
	Sulfolobus tokodaii	-	-	-
**Crenarchaeota/Thermoproteales**	Caldivirga maquilingensis	-	-	-
	Pyrobaculum aerophilum	-	-	-
	Pyrobaculum arsenaticum	-	-	-
	Pyrobaculum calidifontis	-	-	-
	Pyrobaculum islandicum	-	-	-
	Thermofilum pendens	-	-	-
**Euryarchaeota/Archaeoglobales**	Archaeoglobus fulgidus	-	-	-
**Euryarchaeota/Halobacteriales**	Haloarcula marismortui	+C	-	-
	Halobacterium sp.	-	-	-
	Haloquadratum walsbyi	-	-	-
	Halorubrum lacusprofundi	-	-	-
	Natronomonas pharaonis	-	-	-
**Euryarchaeota/Methanobacteriales**	Methanobrevibacter smithii	-	-	-
	Methanosphaera stadtmanae	-	-	-
	Methanothermobacter thermautotrophicus	-	-	-
**Euryarchaeota/Methanococcales**	Methanocaldococcus jannaschii	+U	+	+

	Methanococcus aeolicus	+U	+	+
	Methanococcus maripaludis C5	+U	+	+
	Methanococcus maripaludis C7	+U	+	+
	Methanococcus maripaludis S2	+U	+	+
	Methanococcus vannielii	+U	+	+
**Euryarchaeota/Methanomicrobiales**	Candidatus Methanoregula boonei	-	-	-
	Methanocorpusculum labreanum	-	-	-
	Methanoculleus marisnigri	-	-	-
	Methanospirillum hungatei	-	-	-
**Euryarchaeota/Methanopyrales**	Methanopyrus kandleri	+U	+	-
**Euryarchaeota/Methanosarcinales**	Methanococcoides burtonii	-	-	-
	Methanosaeta thermophila	-	-	-
	Methanosarcina acetivorans	-	-	-
	Methanosarcina barkeri	-	-	-
	Methanosarcina mazei	-	-	-
**Euryarchaeota/Thermococcales**	Pyrococcus abyssi	-	-	-
	Pyrococcus furiosus	-	-	-
	Pyrococcus horikoshii	-	-	-
	Thermococcus kodakarensis	-	-	-
**Euryarchaeota/Thermoplasmales**	Ferroplasma acidarmanus	-	-	-
	Picrophilus torridus	-	-	-
	Thermoplasma acidophilum	-	-	-
	Thermoplasma volcanium	-	-	-
**Nanoarchaeota**	Nanoarchaeum equitans	-	-	-

Note: +C: Cys-containing SelD
+U: Sec-containing SelD
